# Chinese Herbal Medicine, Guilu Erxian Glue, as Alternative Medicine for Adverse Side Effects of Chemotherapy in Doxorubicin-Treated Cell and Mouse Models

**DOI:** 10.1155/2021/5548968

**Published:** 2021-04-05

**Authors:** Chia-Ying Lien, Chen-Wen Lu, Yi-Hsuan Lin, Wan-Jhen Wu, Chih-Hsiang Hsu, Tai-Yuan Chuang, Kuei-Fu Lin, Wu-Chang Chuang, Ming-Chung Lee, Chung-Hsin Wu

**Affiliations:** ^1^Department of Athletics, National Taiwan University, Taipei, Taiwan; ^2^School of Life Science, National Taiwan Normal University, Taipei, Taiwan; ^3^Department of Physical Education, National Tsing Hua University, Hsinchu, Taiwan; ^4^Sun Ten Pharmaceutical Co. Ltd., New Taipei, Taiwan; ^5^Brion Research Institute of Taiwan, New Taipei, Taiwan

## Abstract

Doxorubicin (DOX), a chemotherapeutic drug, often causes many adverse side effects in patients with cancer, such as weight loss, motor disability, blood circulation defects, myelosuppression, myocardial injury, joint degeneration, and bone loss. The Chinese herbal medicine Guilu Erxian Glue (GEG) has been used in the prevention and treatment of osteoarthritis and osteoporosis for hundreds of years, with considerably fewer side effects. We expected that GEG could serve as a protective and beneficial alternative treatment for DOX-induced adverse side effects. In this study, we evaluated whether GEG can alleviate DOX-induced weight loss, motor disability, abnormal blood circulation, myelosuppression, myocardial injury, joint degeneration, and bone loss by using chemotherapy models of synoviocyte cell line HIG-82 and mice. Moreover, we examined the antioxidant capacity of GEG by using DPPH (1,1-diphenyl-2-picrylhydrazyl) free-radical scavenging. Our results revealed that GEG treatment can significantly enhance DPPH free-radical scavenging and reduce DOX-induced cytotoxicity in synoviocyte HIG-82 cells. In addition, GEG treatment for 2 weeks can significantly relieve weight loss, enhance exhaustive exercise capacity, improve blood circulation, alleviate myocardial oxidative stress and inflammation, and strengthen the tibias of DOX-treated mice. Thus, we suggest that GEG treatment can be a protective and alternative therapy for alleviating chemotherapy-related side effects such as weight loss, motor disability, blood circulation defects, and bone loss.

## 1. Introduction

Patients with cancer undergoing chemotherapy often report many adverse side effects, such as weight loss, motor disability, blood circulation defects, myocardial injury, joint degeneration, and bone loss [1–10]. In addition, chemotherapy causes vomiting, hair loss, anemia, and other symptoms, which makes it difficult to perform activities of daily life and maintain exercise habits [11]. Among chemotherapy drugs, doxorubicin (DOX) is an effective chemotherapeutic agent used in the treatment of human lymphoma, leukemia, and solid tumors [6–10]. However, DOX also causes irreversible heart failure, muscle damage, osteoarthritis, and osteoporosis [1–4]. Oxidative stress and inflammation were reported to be involved in the pathogenesis of DOX-induced cardiotoxicity [12–18]. Increased oxidative stress can reduce mitochondria activity and lead to apoptosis [19, 20]. Our previous study revealed that DOX-treated mice exhibited myocardial oxidative stress, inflammation, and apoptosis, whereas the herbal formula B307 provided cardioprotection in DOX-treated mice through the suppression of oxidative stress, inflammation, and apoptosis [21]. Clinical evidence suggests that DOX induces severe osteoporosis and osteoarthritis in addition to myocardial injury in patients with cancer undergoing chemotherapy [22–27]. For example, children receiving DOX chemotherapy might experience long-term bone damage, leading to reduced adult height and increased fracture risk [22]. Premenopausal patients with breast cancer receiving DOX chemotherapy exhibited significantly low bone density and bone loss [23]. In laboratory experiments, DOX chemotherapy caused a 60% reduction in bone formation in normal rats [24, 25] and a significant reduction in trabecular bone volume and cortical bone thickness in rabbits [26]. At the cellular level, DOX treatment could inhibit osteoblast cell differentiation in mice [27]. Osteoarthritis often presents risks of falls and disability, whereas osteoporosis often leads to high risks of fractures and subsequent complications in older people and patients with cancer undergoing chemotherapy [28–30]. Identifying a potential reliever that can protect from the adverse side effects of chemotherapy without reducing the effectiveness of chemotherapy for patients with cancer receiving DOX treatment is urgent.

Guilu Erxian Glue (GEG) is a typical Chinese herbal medicine used in Taiwan that contains four major components, *Cornu Cervi*, *Testudinis Plastrum*, *Ginseng Radix*, and *Lycii Fructus*. GEG has been widely applied in the treatment and prevention of osteoporosis and osteoporosis for hundreds of years with remarkably few side effects [31]. In addition, GEG treatment has long-term beneficial effects on aging, perimenopausal syndrome, and degenerative joint disease [32]. In vitro, GEG treatment can stimulate the secretion of IGF-1 in osteoblasts and attenuate bone osteoclast reabsorption [33]. In vivo, GEG treatment can inhibit the formation of osteoclasts and bone pits in rats and reduce articular pain and increase muscle strength in elderly men with knee osteoarthritis [34]. Notably, GEG treatment can prevent and treat myelosuppression following cancer chemotherapy [35]. However, whether GEG can alleviate DOX-induced cardiotoxicity, muscle weakness, osteoarthritis, and osteoporosis has not been fully elucidated.

In this study, we mainly investigated the alleviating effects of GEG treatments in DOX-induced cytotoxicity in synoviocyte cell line HIG-82 and on weight loss, motor disability, blood circulation defects, myelosuppression, myocardial injury, and bone loss in mice subjected to chemotherapy. Our results may provide evidence to suggest that GEG treatment is a protective and beneficial alternative therapy for DOX-induced adverse side effects.

## 2. Materials and Methods

### 2.1. Preparation of GEG

GEG (supplied by Sun-Ten Pharmaceutical Company, New Taipei City, Taiwan) contains four major components, *Testudinis Plastrum*, *Cornu Cervi*, *Lycii Fructus*, and *Ginseng Radix*, in the ratios of 4 : 2: 2 : 1. All chemical compounds used in this analysis were dissolved in distilled water (H_2_O)/methanol (MeOH). Chromatographic fingerprint analysis was conducted using a 3D high-performance liquid chromatography (HPLC) that mainly followed the methods of our previous study [36].

### 2.2. DPPH Assay of GEG Treatment

We assessed the antioxidant activities of GEG through a DPPH (1,1-diphenyl-2-picrylhydrazyl) assay. As described in the previous study [37], GEG extract (1–20 mg/mL) diluted in distilled water was mixed with 100 *μ*L of 1.5 mM/mL DPPH (D9132, Sigma-Aldrich Co., St. Louis, MO, USA) in methanol in a 96-well plate. After the samples were left to stand for 30 min at room temperature, their absorbance was recorded. The color changes were recorded spectrophotometrically at 517 nm by using a Microplate Spectrophotometer (*µ*Quant, Biotek Intruments, Inc., VT, USA). Appropriate blanks (methanol) and standards (L-Ascorbic acid in water, L-AA; A5960, Sigma-Aldrich) were recorded simultaneously. Each assay was performed in triplicate. The inhibitory activity (%) of DPPH scavenging was calculated using the following expression. DPPH scavenging (%) = 100 × [(absorbance of sample + DPPH) − (absorbance of sample blank)]/[(absorbance of DPPH) − (absorbance of methanol)]. Concentrations of GEG that cause 50% scavenging (IC_50_) were calculated from the graph in which the scavenging activity was plotted against the corresponding GEG concentration.

### 2.3. MTT Assay of GEG Treatment

We assessed the DOX-induced (D1515, Sigma-Aldrich) cytotoxicity by using the MTT assay on rabbit synoviocyte HIG-82 cells. Synoviocyte HIG-82 cells (ATCC CRL1832) were purchased from the Bioresource Collection and Research Center (BCRC#60242, Hsinchu, Taiwan). The methods of MTT assay used in this study mainly followed the methods of our previous study [38]. After treatment with GEG and DOX, 0.5 mg/mL 3-(4,5-dimethylthiazol-2-yl)- 2,5-diphenyltetrazolium bromide (MTT, M5655, Sigma-Aldrich) was added to the culture media. Absorbance was read at an optical density (OD) of 570 nm with an enzyme-linked immunosorbent assay reader (µQuant, Biotek Intruments, Inc., VT, USA).

### 2.4. Animal Preparation

A total of 32 five-month-old male ICR (Institute of Cancer Research) mice were purchased from the BioLASCO Breeding Center (AAALAC International awarded, Yi-Lan, Taiwan) and used in this study. Animal experiments were permitted and supervised by the Institutional Animal Care and Use Committee of National Taiwan Normal University (Protocol number: NTNU/Animal Use/No. 109006/Mar. 10, 2020). All experiment protocols were executed in accordance with the international guidelines for the Care and Use of Laboratory Animals. The methods of animal preparation and grouping used in this study mainly followed the methods of our previous study [21]. ICR mice were randomly divided into four groups: sham (negative control group), GEG (positive control group), DOX (DOX treatment group), and DOX + GEG (DOX + GEG treatment group). Mice in the sham group were fed a vehicle (dimethyl sulfoxide) through their drinking water twice daily for 14 days. In the GEG group without DOX treatment, mice were fed GEG extract (30 mg/mL, pH close to 7.0) through their drinking water twice daily for 14 days. In the DOX group, before DOX treatment, mice were fed a vehicle (dimethyl sulfoxide) through their drinking water twice daily for 14 days and then treated with two intraperitoneal injections of DOX (10 mg/kg body weight). In the DOX + GEG group, before DOX treatment, mice were fed GEG extract (30 mg/mL, pH close to 7.0) through their drinking water twice daily for 14 days and then treated with two intraperitoneal injections of DOX (10 mg/kg body weight). Our animal experiments were considered to be in accordance with the 3R principles (replace, reduce, and refine) for optimizing the experimental design.

### 2.5. Exhaustive Swimming Experiment Design

We assessed the exhaustive exercise capacity by conducting the exhaustive swimming experiment in all mice. Sham, GEG-, DOX-, and DOX + GEG-treated mice were subjected to a weight-loaded exhaustive swimming procedure in a swimming tank (50 × 50 × 50 cm^3^) with 30 cm-deep water maintained at 25 ± 3°C for 1 h. The methods of exhaustive swimming experiment used in this study mainly followed the methods of our previous study [36].

### 2.6. Subcutaneous Microcirculation Measurement

We assessed the blood circulation function in all mice through subcutaneous microcirculation measurement. A laser Doppler imager (Moor Instruments, Axminister, UK) was used to scan the regional dermal microvascular blood flow of mice in the sham, GEG, DOX, and DOX + GEG treatment groups individually. The methods of subcutaneous microcirculation measurement used in this study mainly followed the methods of our previous study [39]. We selected and averaged the subcutaneous microcirculation measurements for each mouse obtained from at least three stable consecutive laser Doppler images.

### 2.7. Peripheral Blood Smear Analysis

We assessed the hemogram of all mice by using peripheral blood smear. Blood smear analysis is an integral part of a hemogram because it allows the quantification of different types of leukocytes and detection of morphologic abnormalities that may be indicators of pathophysiological processes. The methods of peripheral blood smear analysis used in this study mainly followed the methods of our previous study [40]. We collected blood from mice in the sham, GEG, DOX, and DOX + GEG treatment groups and conducted subsequent hematological staining with a Romanowsky stain. Blood cell morphology was evaluated in the area of the smear where the red blood cells and white blood cells were counted.

### 2.8. Cardiac Immunohistochemistry

The mice in the sham, GEG, DOX, and DOX + GEG treatment groups were anesthetized and then cardiac-perfused with PBS containing 4% formaldehyde (EM grade glutaraldehyde solution, Sigma-Aldrich). We removed cardiac tissue from the mice and fixed it in 4% formaldehyde. Cardiac specimens were then embedded in paraffin and cut into 5 *μ*m-thick tissue sections. Then, tissue sections were mounted on slides for histological and immunohistochemical (IHC) analysis. The methods of cardiac immunohistochemistry used in this study mainly followed the methods of our previous study [21].

### 2.9. Tibia Micro-CT Measurement

We assessed the bone density of the tibia in all mice through micro-CT measurement, which was performed by the Taiwan Mouse Clinic. A Skyscan 1076 micro-CT device (Skyscan, Aartselaar, Belgium) was used to obtain the micro-CT images of the tibias of the mice in the sham, GEG, DOX, and DOX + GEG treatment groups. The scanning parameters were set as follows: 49 kV, 200 mA, 500 ms, and a voxel resolution of 18.27 mm. The micro-CT images were imported into CTAn software (Skyscan), and the bone volume of the tibias was calculated using ImageJ (Rasband, W.S., ImageJ, U.S. National Institutes of Health, Bethesda, MD, USA).

### 2.10. Statistical Analysis

The methods of statistical analysis used in this study mainly followed the methods of our previous study [39]. We expressed data as the mean ± standard error of the mean (SEM). Differences among the sham, GEG, DOX, and DOX + GEG treatment groups were evaluated using two-way analysis of variance (ANOVA). The Student–Newman–Keuls multiple comparison post hoc test was performed if a significant F value was obtained. Significance was defined as *p* < 0.05.

## 3. Results

### 3.1. Chromatographic Fingerprint of GEG

**Figure 1:**
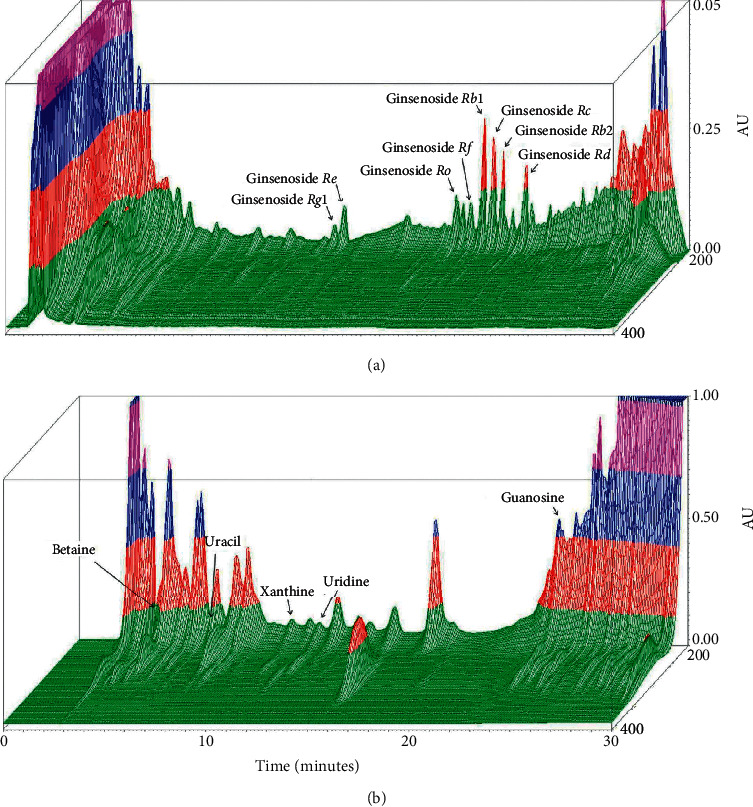
Chromatographic fingerprint analysis for the herbal formula Guilu Erxian Glue (GEG). GEG is a combination of traditional Chinese medicine with four main ingredients of ginseng (*Radix Ginseng*), wolfberry (*Fructus Lycii*), tortoise plastron (*Carapax et Plastrum Testudinis*), and antler (*Cornu Cervi*) from a famous Chinese prescription. (a) 3D-HPLC fingerprint of Ginsenoside (200–400 nm, 0–65 min). (b) 3D-HPLC fingerprint of Betaine and Nucleosides (200–400 nm, 0–30 min). Chromatographic fingerprint analysis was conducted through the HPLC and LC/MS. Abbreviations: GEG, Guilu Erxian Gum; AU, arbitrary perfusion units; and HPLC, high-performance liquid chromatography.

GEG is a combination of traditional Chinese medicine with four main ingredients of ginseng (*Radix Ginseng*), wolfberry (*Fructus Lycii*), tortoise plastron (*Carapax et Plastrum Testudinis*), and antler (*Cornu Cervi*) from a famous Chinese prescription. Chromatographic fingerprint analysis using LC/MS analysis for bioactive marker substances of the GEG is shown in Figure 1. Bioactive marker substances for Ginsenoside (200–400 nm, 0–65 min) were Ginsenoside Rg1 (27.1 min), Ginsenoside Re (28.1 min), Ginsenoside Ro (40.2 min), Ginsenoside Rf (41.6 min), Ginsenoside Rb1 (43.2 min), Ginsenoside Rc (44.2 min), Ginsenoside Rb2 (45.2 min), and Ginsenoside Rd (47.6 min) (Figure 1(a)) and for Betaine and Nucleosides (200–400 nm, 0–30 min) were Betaine (3.5 min), Uracil (5.7 min), Xanthine (10.4 min), Uridine (11.8 min), and Guanosine (23.2 min) (Figure 1(b)).

### 3.2. DPPH Free-Radical Scavenging Activity of GEG

**Figure 2:**
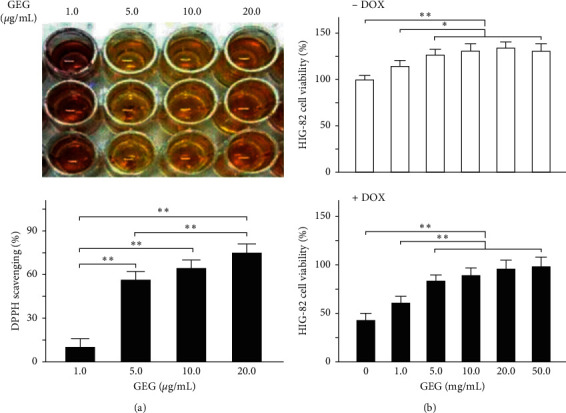
Free-radical scavenging activity and HIG-82 cell viability after GEG treatment. (a) Quantified DPPH radical scavenging activity at various concentrations of GEG (*N* = 3). (b) Quantified relative HIG-82 cell viability at various concentrations of GEG with (+DOX) and without (−DOX) DOX treatment (*N* = 3 for each). Values are expressed as the mean ± SEM (^*∗∗*^*p* < 0.01, ^*∗*^*p* < 0.05, two-way ANOVA followed by a Student–Newman–Keuls multiple-comparison posttest). Abbreviations: GEG, Guilu Erxian Gum; DPPH, 1,1-diphenyl-2-picrylhydrazyl; DOX: doxorubicin; SEM, standard error of the mean; and ANOVA, analysis of variance.

The free-radical scavenging activity of GEG extract at various concentrations was measured, and the results are depicted in Figure 2(a). Significant DPPH radical scavenging activity was evident at GEG concentrations of 5–20 mg/mL. Moreover, treatment with 5–20 mg/mL GEG extract yielded superior antioxidant activity (55.2%–72.7%) compared with treatment with 1 mg/mL GEG extract (10.1%). The quantified DPPH free-radical scavenging activity was similar for 10 and 20 mg/mL GEG extract; however, significant free-radical scavenging activity was observed at 20 mg/mL GEG extract treatment (*p* < 0.01).

### 3.3. Effect of GEG Treatment on the Cell Viability of DOX-Treated Synoviocyte HIG-82 Cells

Quantified relative values of percentage cell viability of the HIG-82 cells at different concentrations of GEG extract with and without DOX treatment after 48 h incubation are shown in Figure 2(b). The HIG-82 cell viability at concentrations of 5–50 mg/mL of GEG extract without DOX treatment differed significantly from that of the control (0 mg/mL of GEG extract without DOX treatment). Moreover, treatment with 1–50 mg/mL GEG extract without DOX treatment yielded higher cell viability (115%–131%) compared with treatment with 0 mg/mL GEG extract without DOX (100%). Furthermore, our data revealed that the cell viability of HIG-82 cells was significantly reduced to approximately 56% after DOX treatment with 0 mg/mL GEG extract (*p* < 0.01), whereas the cell viability of DOX-treated HIG-82 cells was significantly increased to 61%–99% at GEG concentrations of 1–50 mg/mL. The cell viability was significantly different (*p* < 0.01) at concentrations of 5–50 mg/mL GEG extract with DOX treatment than at concentrations of 0 and 1 mg/of GEG extract with DOX treatment.

### 3.4. Effect of GEG Treatment on Weight Loss in DOX-Treated Mice

**Figure 3:**
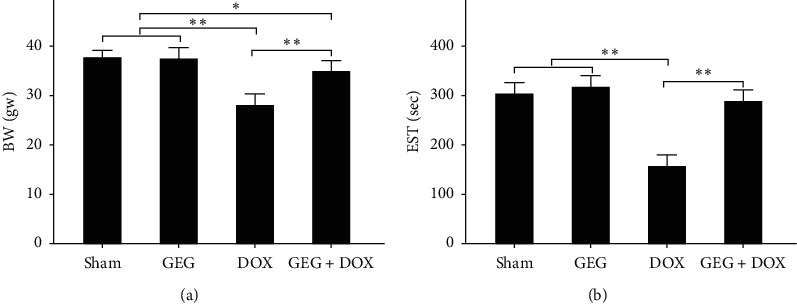
Effects of GEG treatment on the body weight and exhaustive swimming time of mice with and without DOX treatment. (a) Comparison of quantified body weight among the mice treated with sham, GEG, DOX, and DOX + GEG (*N* = 8 for each group). (b) Comparison of EST among mice treated with sham, GEG, DOX, and DOX + GEG (*N* = 8 for each group). Values are expressed as the mean ± SEM (^*∗∗*^*p* < 0.01, ^*∗*^*p* < 0.05, two-way ANOVA followed by a Student–Newman–Keuls multiple comparison posttest). Abbreviations: GEG, Guilu Erxian Gum; EST, exhaustive swimming time; DOX, doxorubicin; SEM, standard error of the mean; ANOVA, analysis of variance.

The quantified body weights of the mice in the sham, GEG, DOX, and DOX + GEG treatment groups are presented in Figure 3(a). No significant difference in body weight was observed between mice treated with sham and those treated with GEG (sham, 38.1 gw vs. GEG, 38.0 gw; *p* > 0.05). The body weights of the mice in the GEG treatment group after DOX treatment were significantly reduced compared with those of the mice in the sham treatment group (DOX, 28.5 gw vs. sham, 38.1 gw, *p* < 0.01). Moreover, the weight loss of DOX-treated mice was significantly mitigated after GEG treatment (DOX, 28.5 gw vs. DOX + GEG, 35.3 gw, *p* < 0.01).

### 3.5. Effect of GEG Treatment on the Exercise Capacity of DOX-Treated Mice

The quantified exhaustive swimming times of the mice in the sham, GEG, DOX, and DOX + GEG treatment groups are shown in Figure 3(b). Similar to the changes in body weight, no significant difference in exhaustive swimming time was observed between mice treated with sham and those treated with GEG (sham, 307 s vs. GEG, 320 s, *p* > 0.05). The exhaustive swimming time was significantly reduced in DOX-treated mice compared with that in sham-treated mice (DOX, 160 s vs. sham, 307 s, *p* < 0.01). However, the exhaustive swimming time was significantly increased in DOX-treated mice receiving oral GEG treatment compared with DOX-treated mice that were not treated with GEG (DOX + GEG, 292 s vs. DOX, 160 s, *p* < 0.01).

### 3.6. Effect of GEG Treatment on Subcutaneous Microcirculation in DOX-Treated Mice

**Figure 4:**
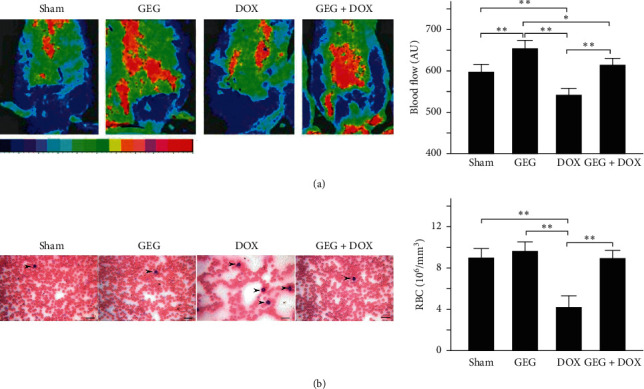
Effects of GEG treatment on subcutaneous microcirculation and RBC count in mice treated with and without DOX. (a) Dorsal imaging of subcutaneous microcirculation performed using moorFLPI laser Doppler imaging (left) and comparison of quantified subcutaneous blood flow (right) among mice treated with sham, GEG, DOX, and DOX + GEG (*N* = 8 for each group). (b) Peripheral blood smear analysis (left) and comparison of the quantified number of RBCs (right) among mice treated with sham, GEG, DOX, and DOX + GEG (*N* = 8 for each group). WBCs are marked with arrows. Values are expressed as the mean ± SEM (^*∗∗*^*p* < 0.01, ^*∗*^*p* < 0.05, one-way ANOVA followed by a Student–Newman–Keuls multiple-comparison posttest). Abbreviations: AU, arbitrary unit; RBC: red blood cell; WBC: white blood cell; GEG, Guilu Erxian Gum; DOX, doxorubicin; SEM, standard error of the mean; and ANOVA, analysis of variance.

Dorsal images of subcutaneous microcirculation and quantified subcutaneous blood flow among the mice in the sham, GEG, DOX, and DOX + GEG treatment groups are shown in Figure 4(a). The subcutaneous microcirculation of mice treated with GEG was obviously enhanced but was obviously lower following DOX treatment. The quantification results revealed that the subcutaneous circulation of mice was significantly enhanced under GEG treatment (sham, 202 AU vs. GEG, 257 AU, *p* < 0.01), but was significantly reduced under DOX treatment (sham, 202 AU vs. DOX, 143 AU, *p* < 0.01). Compared with the DOX-treated mice without GEG treatment, the DOX-treated mice with GEG treatment exhibited significantly enhanced subcutaneous circulation (DOX + GEG, 218 AU vs. DOX, 143 AU, *p* < 0.01).

### 3.7. Effect of GEG Treatment on Red Blood Cell Count in DOX-Treated Mice

Peripheral blood smear analysis is routinely performed in our laboratory to evaluate myelosuppression related to dysfunction in blood cell production. We evaluated the peripheral blood smear of mice treated with sham, GEG, DOX, and DOX + GEG, and the results are presented in Figure 4(b). We observed that the number of red blood cells was obviously decreased but that the number of white blood cells was obviously increased in mice following DOX treatment. Quantification of the number of red blood cells revealed no significant difference between the mice treated with sham and those treated with GEG (sham, 9.4 × 10^6^/mm^3^ vs. GEG 9.6 × 10^6^/mm^3^, *p* > 0.05); by contrast, the red blood cell count was significantly reduced in DOX-treated mice (sham, 9.4 × 10^6^/mm^3^ vs. DOX, 4.3 × 10^6^/mm^3^, *p* < 0.01). Two weeks after oral GEG treatment, the number of red blood cells was significantly increased in DOX-treated mice (DOX, 4.3 × 10^6^/mm^3^ vs. sham, 9.1 × 10^6^/mm^3^, *p* < 0.01).

### 3.8. Effect of GEG Treatment on Cardiac Oxidative Stress and Inflammation in DOX-Treated Mice

**Figure 5:**
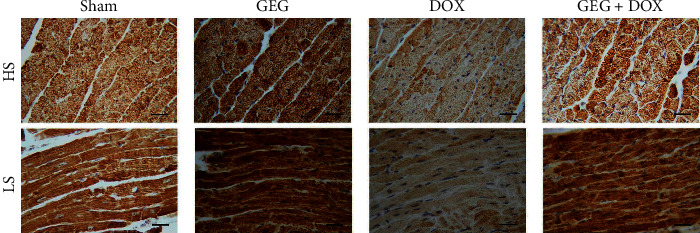
Effects of GEG treatment on antioxidant-stress-related SOD2 expression in the myocardial tissue of mice treated with and without DOX. Horizontal and longitudinal cross sections of IHC staining results showing myocardial SOD2 expression in mice treated with sham, GEG, DOX, and DOX + GEG. Bar scale = 30 *μ*m. Abbreviations: HS, horizontal cross sections; LS, longitudinal cross sections; SOD2, superoxide dismutase 2; GEG, Guilu Erxian Gum; and DOX, doxorubicin.

**Figure 6:**
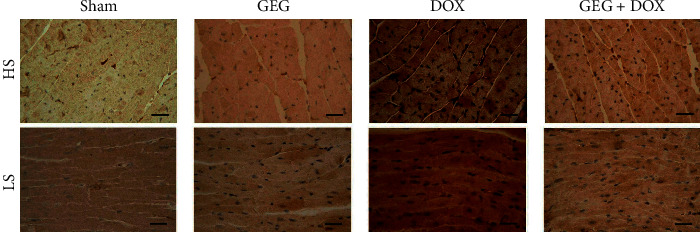
Effects of GEG treatment on inflammation-related TNF-*α* expression in the myocardial tissue of mice with and without DOX treatment. Horizontal and longitudinal cross sections of IHC staining showing myocardial TNF-*α* expression in mice treated with sham, GEG, DOX, and DOX + GEG. Bar scale = 30 *μ*m. Abbreviations: HS, horizontal cross sections; LS, longitudinal cross sections; TNF-*α*, tumor necrosis factor alpha; GEG, Guilu Erxian Gum; and DOX, doxorubicin.

We evaluated cardiac oxidative stress by using IHC staining for SOD2 in the cardiac tissues of mice treated with sham, GEG, DOX, and DOX + GEG, and the results are presented in Figure 5. SOD2 plays a major role in defense against free radicals. We observed that cardiac SOD2 expression was obviously enhanced in mice treated with GEG but was obviously reduced in mice following DOX treatment. Compared with DOX-treated mice that were not treated with GEG, those that were treated with GEG exhibited noticeably increased cardiac SOD2 expression levels. Irrespective of the presence or absence of DOX treatment, GEG can obviously relieve cardiac oxidative stress in mice. Furthermore, we evaluated inflammation through IHC staining of TNF-*α* in the cardiac tissue of mice treated with sham, GEG, DOX, and DOX + GEG, and the results are presented in Figure 6. TNF-*α* is a strong proinflammatory cytokine that plays an important role in the immune system during inflammation. We observed that the cardiac TNF-*α* expression levels of mice treated with GEG did not exhibit obvious changes; however, the expression levels were obviously enhanced in mice following DOX treatment. Compared with DOX-treated mice that were not treated with GEG, those that were treated with GEG exhibited noticeably reduced cardiac TNF-*α* expression levels.

### 3.9. Effect of GEG Treatment on Tibia Bone Density in DOX-Treated Mice

**Figure 7:**
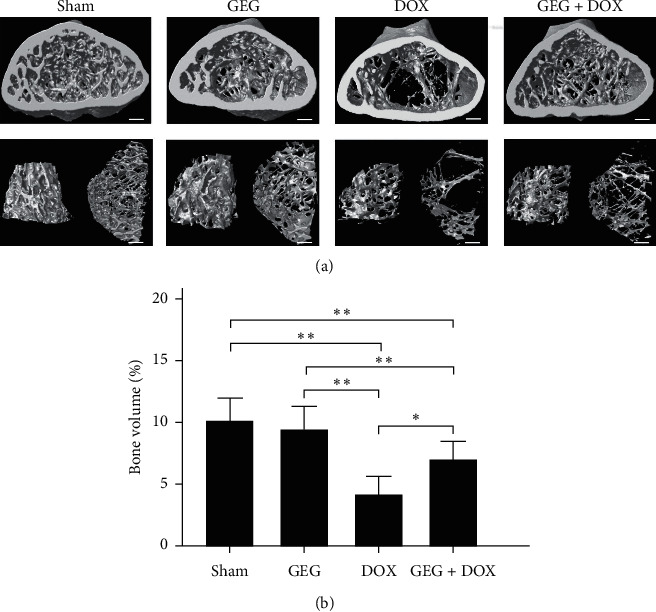
Effects of GEG treatment on bone density in the tibia tissue of mice with and without DOX treatment. (a) Cross-sectional imaging of the tibia and trabecular bone among mice treated with sham, GEG, DOX, and DOX + GEG performed by high-resolution microcomputed tomography. Bar scale = 100 *μ*m. (b) Comparison of the quantified bone volume percentage of the tibia among mice treated with sham, GEG, DOX, and DOX + GEG (*N* = 3 for each group). Values are expressed as the mean ± SEM (^*∗∗*^*p* < 0.01, ^*∗*^*p* < 0.05, one-way ANOVA followed by a Student–Newman–Keuls multiple-comparison posttest). Abbreviations: GEG, Guilu Erxian Gum; DOX, doxorubicin; SEM, standard error of the mean; and ANOVA, analysis of variance.

High-resolution microcomputed tomography was employed for cross-sectional imaging of the tibias and trabecular bones of the mice treated with sham, GEG, DOX, and DOX + GEG (Figure 7(a)). We observed that the tibia and trabecular bone density in the mice treated with sham and GEG was considerably high but obviously low in the DOX-treated mice. The density of the tibia and trabecular bone in DOX-treated mice after oral GEG treatment was obviously increased compared with that of the DOX-treated mice that were not treated with GEG. We quantified the bone density of the mice treated with sham, GEG, DOX, and DOX + GEG, and the results are presented in Figure 7(b). No significant difference in bone density was observed between the mice treated with sham and those treated with GEG (sham, 9.9% vs. GEG, 9.7%, *p* > 0.05). The bone density of the mice treated with DOX was significantly reduced compared with that of the mice treated with sham (DOX, 4.2% vs. sham, 9.9%, *p* < 0.01). However, bone loss was significantly mitigated in DOX-treated mice after GEG treatment (DOX, 4.2% vs. DOX + GEG, 7.1%, *p* < 0.01). Although the DOX-treated mice receiving GEG treatment could not return to their normal bone density levels (sham, 9.9% vs. GEG + DOX, 7.1%, *p* < 0.01), the results indicate that GEG treatment significantly increased the bone density in mice after DOX treatment.

## 4. Discussion

In the present study, we used DOX, a chemotherapeutic drug, to treat the synoviocyte cell line HIG-82 and mice to evaluate whether GEG can be used in the prevention and treatment of chemotherapy-induced side effects such as weight loss, motor disability, myocardial injury, joint degeneration, and bone loss. GEG is a multicomponent formula that has been widely used in the treatment and prevention of osteoporosis for hundreds of years. GEG contains *Cornu Cervi*, *Testudinis Plastrum*, *Ginseng Radix*, and *Lycii Fructus* in specific ratios. *Cornu Cervi* and *Testudinis Plastrum* can alleviate fatigue and increase red blood cell counts and hemoglobin levels. *Ginseng Radix* can strengthen immunity and hematopoietic function, alleviate fatigue, and strengthen the heart. *Lycii Fructus* has nourishing properties and can improve eyesight, promote liver cell regeneration, increase hematopoietic function, and strengthen immunity [32]. The ancient Chinese herbal medicine book “*Compendium of Materia Medica*” reported that *Cornu Cervi* can promote bone growth and prevent osteoporosis, thereby nourishing the yang. *Testudinis Plastrum* can strengthen the heart, kidney, and blood, thereby nourishing the yin. If *Cornu Cervi*, *Testudinis Plastrum*, *Ginseng Radix*, and *Lycii Fructus* function as expected, people can achieve strength and vitality by consuming the four ingredients in one formula [31]. The present study compared the effects of GEG treatment on DPPH free-radical scavenging; cytotoxicity in DOX-treated synoviocytes; and weight loss, exhaustive exercise capacity, myocardial oxidative stress and inflammation, and tibia bone density in DOX-treated mice. The major findings of this study reveal that GEG can significantly enhance DPPH free-radical scavenging (Figure 2(a)) and reduce DOX-induced cytotoxicity in synoviocytes (Figure 2(b)). In addition, the results indicate that GEG treatment can significantly mitigate weight loss (Figure 3(a)), enhance exhaustive exercise capacity (Figure 3(b)), improve blood circulation (Figure 4(a)), alleviate myocardial oxidative stress (Figure 5) and inflammation (Figure 6), and increase tibia bone density (Figure 7) in DOX-treated mice. To the best of our knowledge, the present study is the first to conduct an evidence-based investigation of the effectiveness of alternative therapy with the traditional Chinese medicine GEG in reversing DOX-induced weight loss, motor disability, blood circulation defects, myocardial injury, joint degeneration, and bone loss.

DOX is an effective anticancer agent that inhibits cell proliferation but induces oxidative stress, ultimately leading to cell death, mainly through apoptosis [41]. Free radicals are mainly produced in the mitochondria, and they increase oxidative stress. Reactive oxygen species (ROS) comprise free radicals that can be permanently removed by a complex antioxidant system. Imbalance in the antioxidant system may cause oxidative stress and subsequent ROS overproduction. The present study demonstrated that GEG treatment significantly enhanced DPPH free-radical scavenging activity (Figure 2(a)) and alleviated myocardial oxidative stress by enhancing the cardiac expression of SOD2 (Figure 5). Consistent with our previous report [21], we found that DOX-treated mice exhibited significantly reduced mortality rate, body weight, and cardiac function. In addition, the cardiac expression of endothelial nitric oxide synthase, SOD2, and B-cell lymphoma 2 (Bcl-2) was significantly suppressed, but the expression of TNF-*α*, Bcl-2-associated X protein, calpain, caspase 12, caspase 9, and caspase 3 was significantly enhanced in DOX-treated mice. These results indicate that DOX-treated mice exhibited myocardial oxidative stress, inflammation, and apoptosis. The present study further verified that GEG treatment can mitigate weight loss (Figure 3(a)) and enhance subcutaneous microcirculation (Figure 4(a)). GEG treatment can also alleviate myocardial inflammation by reducing the cardiac expression of TNF-*α* (Figure 6).

In patients with cancer undergoing chemotherapy, ameliorating chemotherapy-induced side effects such as fatigue and muscle weakness is crucial for improving patients' quality of life [42, 43]. Chemotherapy-induced fatigue and muscle weakness seem to be associated with higher cancer mortality [44–46]. For patients undergoing chemotherapy, exercise is considered an effective treatment strategy because it can enhance muscle strength, improve quality of life, and reduce fatigue levels [47]. Additionally, the present study showed that DOX impaired the exhaustive exercise capacity of mice but that GEG treatment significantly improved the muscle strength in DOX-treated mice (Figure 3(b)). GEG has been reported to prevent and treat myelosuppression following cancer chemotherapy [35]. Blood cell deficiency caused by bone marrow suppression is one of the most serious side effects of chemotherapy. Severe bone marrow suppression and hematological toxicity after chemotherapy are major contributors to high mortality and morbidity in patients with cancer. We observed that although the number of red blood cells was obviously reduced, after 2 weeks of oral GEG treatment, the number significantly increased in DOX-treated mice (Figure 4(b)). Our results suggest that adequate exercise plus GEG treatment might be an effective therapy for alleviating chemotherapy-induced adverse side effects such as motor disability and myelosuppression.

Osteoarthritis is the most prevalent form of joint disease and often causes falls, disabilities, and dependency in older people [28, 29]. In vitro experiments with synoviocyte HIG-82 cells isolated from soft tissue lining the knee joints of rabbits are recommended to evaluate the pathophysiology of various arthritides [48]. Our data showed that the viability of HIG-82 cells was significantly reduced to approximately 56% after DOX treatment, whereas the viability of DOX-treated HIG-82 cells was significantly increased to 61%–99% after GEG treatment (Figure 2(b)). The results indicate that DOX treatment may cause synoviocyte damage and even lead to arthritides, whereas GEG treatment may reduce DOX-induced cytotoxicity and facilitate synoviocyte proliferation. In addition, osteoporosis has become a public health concern as a result of the high risk of fractures and subsequent complications that it incurs [30]. Children receiving DOX chemotherapy may experience bone damage in the form of reduced adult height and increased fracture risk [22]. Patients with cancer receiving DOX chemotherapy may exhibit low bone mineral density and significant bone loss [23]. DOX chemotherapy may cause a 60% reduction in bone formation in normal rats [24,25] and reduce trabecular bone volume and cortical bone thickness in rabbits [26]. Our results reveal that the density of the tibia and trabecular bone was obviously decreased in mice treated with DOX, whereas it was obviously increased in DOX-treated mice treated with oral GEG (Figure 7). GEG primarily originates from processed tortoise shells and antlers and has been widely used in China for the treatment and prevention of osteoporosis for hundreds of years with minimal side effects [31]. Our results suggest that DOX treatment may cause osteoporosis in mice but that GEG treatment can relieve this osteoporosis. Although bone density of GEG group mice seems to be lower than that of the sham group, there is no significant difference between sham and GEG group mice (Figure 7(b)). We were worried that long-term oral administration of GEG may reduce bone density. Until now, there is no evidence that long-term oral administration of GEG may cause a crisis of osteoporosis. The mechanisms by which GEG treatment relieves osteoporosis and osteoarthritis remain unclear. Therefore, further clinical trials are warranted to verify the benefits of GEG treatment in chemotherapy-treated patients.

## 5. Conclusions

The study findings reveal that DOX chemotherapy can induce cytotoxicity in synoviocyte HIG-82 cells and cause motor disability, myocardial oxidative stress and inflammation, and bone loss in mice. GEG treatment can significantly enhance DPPH free-radical scavenging activity and alleviate DOX-induced cytotoxicity in synoviocyte HIG-82 cells. Moreover, oral GEG treatment for 2 weeks can significantly mitigate weight loss, enhance exhaustive exercise capacity, alleviate myocardial oxidative stress and inflammation, and strengthen the tibia in DOX-treated mice. Thus, GEG treatment is suggested to be an alternative therapy for alleviating chemotherapy-induced adverse side effects.

## Data Availability

Blood biochemical analysis, DPPH assay, MTT assay, immunohistochemistry, and micro-CT measurement data used to support the findings of this study are included within the article and available from the corresponding author upon request.

## Abbreviations

CT: Computed tomography
DOX: Doxorubicin
DPPH: 1,1-Diphenyl-2-picrylhydrazyl
GEG: Guilu Erxian Glue
IHC: Immunohistochemistry
LC/MS: Liquid chromatography/mass spectrometry
MTT: 3-(4,5-Dimethylthiazol-2-yl)-2,5-diphenyltetrazolium bromide
SEM: Standard error of the mean
SOD2: Superoxide dismutase 2.
